# Cellular DNA content and prognosis in surgically treated squamous carcinoma of the larynx.

**DOI:** 10.1038/bjc.1991.221

**Published:** 1991-06

**Authors:** L. D. Cooke, T. G. Cooke, G. Forster, T. R. Helliwell, P. M. Stell

**Affiliations:** Department of Surgery and Otorhinolaryngology, University of Glasgow, UK.


© Macmillan Press Ltd., 1991

### Cellular DNA content and prognosis in surgically treated squamous carcinoma of the larynx

L.D. Cooke[1], T.G. Cooke[1], G. Forster[1], T.R. Helliwell[2] & P.M. Stell[3]

[1]*Department of Surgery and Otorhinolaryngology, University of Glasgow, Glasgow Royal Infirmary, Glasgow;* [2]*Department of Pathology, University of Liverpool, Royal Liverpool Hospital, Liverpool; and* [3]*Department of Otorhinolaryngology, University of Liverpool, Royal Liverpool Hospital, Liverpool, UK.*

There have been 26 previous series reported of ploidy in squamous cell carcinoma of the head and neck. Of these 12 do not discuss survival (Danes *et al.*, 1987; Ensley *et al.*, 1989; Feinmesser *et al.*, 1990; Franzen *et al.*, 1987*a,b*; Graessel-Pietrusky & Hornstein, 1982; Hemmer & Kreidler, 1990; Johnson *et al.*, 1985; Kaplan *et al.*, 1986; Kearsley *et al.*, 1990; Olinici & Caluser, 1987; Wilson *et al.*, 1988). Of the remaining 14 articles, four do not state the type of treatment the patient received (Boecking *et al.*, 1985; Chen, 1989; Lampe *et al.*, 1987; Oloffson *et al.*, 1986). In seven reports the patients were treated by a variety of combinations of radiotherapy, chemotherapy or surgery (Feichter *et al.*, 1987; Goldsmith *et al.*, 1986; Goldsmith *et al.*, 1987; Guo *et al.*, 1989; Holm, 1982; Sickle-Santanello *et al.*, 1986; Tytor *et al.*, 1987).

Tytor *et al.* (1989) reported that aneuploid tumours of the oral cavity were more likely to respond to preoperative radiotherapy. Kokal *et al.* (1988) found, in a series of 76 patients treated initially by surgery, that patients with diploid tumours fared better. They did not state whether their patients had postoperative radiotherapy, but the wording of their article suggests that they did. Furthermore the patients in their series had tumours at various different sites and it is well known that survival varies widely between different sites. However they allowed for this by multivariate analysis.

We have previously reported that patients with end-stage disease submitted to chemotherapy trials do better if they have an aneuploid tumour (Cooke *et al.*, 1990).

Thus there has been no report of a large number of patients with tumours at one site treated in a similar fashion. We report a relatively large series of patients with a tumour of one site (the larynx) of similar stages (stages III and IV), all submitted to surgery.

### Patients

This report is based on 1,128 patients with a laryngeal tumour seen personally by one of us (PMS) between 1963 and 1990. These patients have been treated throughout by a uniform policy of radiotherapy for $T_1$–$T_3N_0$ tumours not causing stridor, and surgery for patients with palpable lymph node metastases, advanced tumours ($T_4$) and patients with stridor. Two hundred and ninety-eight patients with a previously untreated squamous carcinoma were treated initially by surgery. Unfortunately too little histological material was available from specimens of patients seen before 1978. Histological blocks containing enough material for flow cytometry were still available on 110 patients seen since 1978, and these form the basis of this report.

Correspondence: P.M. Stell, Department of Otorhinolaryngology, University of Liverpool, Royal Liverpool Hospital, PO Box 147, Liverpool L69 3BX, UK.
Received 15 October 1990; accepted 17 January 1991.

### *Storage of the data and follow-up*

The data on all these patients have been recorded prospectively, initially on cards, and for the last 10 years on a microprocessor. Data have been kept up to date by personal contact, and by information from general practitioners, the Mersey Regional Cancer Registry, and the National Health Service Register. Two patients (2%) have been lost to follow-up.

### *Staging*

The TNM stage of all patients was classified by the UICC (1987) convention, with appropriate stage grouping.

### *General condition*

The patients' performance status was recorded by the ECOG classification (Beahrs *et al.*, 1988).

### Method

### *DNA measurement and classification*

Thick sections from tumours were examined by flow cytometry, consecutive 5 µm sections being stained by haematoxlin and eosin to confirm the presence of tumour in all samples studied. Briefly, nuclei were extracted from formalin fixed paraffin embedded tissue by the method described by Hedley *et al.* (1983). Multiple 50 µm sections were dewaxed in xylene and rehydrated through 0.5% pepsin in 0.9% NaCl with a pH 1.5 for 30 min at 37°C. The digest was then centrifuged, washed and resuspended. After resuspension in 1 ml of phosphate buffered saline the digest was syringed 3–4 times to disaggregate nuclear clumps and then filtered through 40 µm nylon mesh. Nuclear concentrations were adjusted when necessary to give a final concentration of $10^6$ nuclei per ml. DNA analysis was performed using a Profile-II flow cytometer (Coulter Corp. Hialeh, Florida, USA). Where possible fluorescence from 100,000 nuclei was recorded, a minimum of 10,000 being required to give interpretable histograms. Histograms were classified as aneuploid or diploid, and only those with a coefficient of variation of less than 8% being accepted. Tetraploid tumours, especially if they represent a small fraction of the whole section, may be difficult to detect as the G0 and G1 of these tumour cells have the same DNA content as normal cells in G2 and M. A 4c peak accounting for more than 15% of the whole cell population was designated aneuploid as it is unlikely that normal cells would have such a high G2/M peak.

### Analysis of the data

Qualitative data are displayed in contingency tables, and analysed by $\chi^2$. The relation between ploidy and host and tumour factors was analysed by weighted logistic regression.

Survival curves were drawn up by the life table method (Armitage, 1987). Differences between survival curves were analysed by multivariate regression analysis (Cox, 1972).

### Results

### *Ploidy and host/tumour factors*

The relation between ploidy and the various host and tumour factors is shown in Tables I and II. There was no statistically significant difference between the diploid and aneuploid tumours with respect to host factors. As regards tumour factors, diploid tumours had a higher proportion of Stage IV tumours, but not significant so, whereas aneuploid tumours were more likely to arise from the supraglottic area and be poorly differentiated.

Weighted logistic regression confirmed that neither sex (z = 0.02) nor age (z = 0.56) nor general condition (z = 0.45) were significantly associated with ploidy. However supraglottic tumours were significantly more likely to be aneuploid (z = 2.11, $P<0.05$), even more so if they were poorly differentiated (z = 2.47, $P<0.025$). However histological grade (z = 0.43) and stage (z = 1.51) were not independent indicators of ploidy.

### *Survival*

The 5 year adjusted survival was 50% for diploid tumours, and 48% for aneuploid tumours. A direct comparison of these two rates is not valid because of the differing incidence of sites, histological grade and stage group. However, Cox's multivariate regression showed that there was no statistically significant difference between survival rates for diploid and aneuploid tumours when these variable factors are taken into account (z = 0.63). Indeed there was no significant overall regression in this group of patients when they were analysed for all known prognostic factors ($\chi^2_4 = 10.89$, $P + 0.09$).

### *Node metastases*

Thirty-nine patients later developed a lymph node metastasis: 26 were submitted to surgery, eight were untreated and five had palliative radiotherapy or chemotherapy. The incidence of histologically proven later lymph node recurrence was 31% for diploid tumours, and 45% for aneuploid tumours (at 3 years). However, Cox's regression analysis showed that ploidy was not a significant predictor of later node recurrence (z = 1.00).

### Discussion

In brief this series shows that ploidy was significantly related to subsite within the larynx: supraglottic tumours were more likely to be aneuploid, particularly if they were poorly differentiated. However ploidy was not related to any other tumour factor (stage or histological grade) nor to host factors (age, sex and general condition). Secondly, we found that ploidy did not affect survival once confounding by the site effect referred to above was taken into account.

We found a higher incidence of nodal metastases in aneuploid tumours, but this was not significant. The difference could be due to the fact that aneuploid tumours are more likely to be supraglottic and the latter tumours are more likely to metastasise as the supraglottis has a well developed external lymphatic drainage.

Our findings are at odds with some investigators, but many published series have been small – the six series referred to in the introduction contained only 157 patients in all, whereas our series contained 110 patients. Furthermore the authors of the above series did not use multivariate methods to assess the relation of ploidy with host and tumour factors, and only one (Kokal *et al.*) used multivariate analysis of survival to allow for confounding. A meta analysis of all the reports of squamous carcinoma of the head and neck published to date shows that tumour DNA analysis has no prognostic significance in laryngeal cancer, though it probably does in mouth cancer (Stell, 1991). Finally, unlike all previous series, our is homogeneous with respect to site, stage of disease and treatment.

The authors were grateful to the CRC and the NWCRF for technical support and to Mrs B. Cowley and Mrs J. Deeprose who did the typing.

**Table I** Ploidy and host factors

| | Ploidy | |
|---|---|---|
| | *Diploid* | *Aneuploid* |
| *Sex* | | |
| Men | 45 | 37 |
| Women | 15 | 13 |
| | $\chi^2_1 = 0.01$ | |
| *Age* | | |
| Mean (in years) | 60.2 | 59.3 |
| *ECOG status* | | |
| O | 43 | 34 |
| I-IV | 15 | 16 |
| Not recorded | 2 | 0 |
| | $\chi^2_1 = 0.23$ | |

**Table II** Ploidy and tumour factors

| | *Diploid* | *Aneuploid* |
|---|---|---|
| *Site* | | |
| Supraglottic | 27 | 38 |
| Glottic | 9 | 5 |
| Subglottic | 12 | 0 |
| Transglottic | 12 | 7 |
| | $\chi^2_3 = 15.5$, $P<0.01$ | |
| *Histology* | | |
| Well differentiated | 13 | 3 |
| Moderately differentiated | 20 | 19 |
| Poorly differentiated | 25 | 27 |
| Ungraded | 2 | 1 |
| | $\chi^2_2 = 5.63$, $P = 0.07$ | |
| *Stage grouping* | | |
| I | 0 | 0 |
| II | 0 | 0 |
| III | 31 | 30 |
| IV | 29 | 20 |
| | $\chi^2_1 = 0.46$, N.S. | |

### References

ARMITAGE, P. (1987). *Statistical Methods in Medical Research*. 2nd edition. Blackwell Scientific Publications: Oxford, p. 421.

BEAHRS, O.H., HENSON, D.E., HUTTER, R.V.P. & MYERS, M.H. (1988). *Manual for Staging of Cancer*, 3rd edition. Lippincott: Philadelphia.

BOECKING, A., AUFFERMANN, W., VOGEL, H., SCHLONDORFF, G. & GOEBBELS, R. (1985). Diagnosis and grading of malignancy in squamous epithelial lesions of the larynx with DNA cytophotometry. *Cancer*, **56**, 1600.

CHEN, R. (1989). Flow cytometric analysis of benign and malignant tumours of the oral and maxillofacial region. *J. Oral Maxillofac. Surg.*, **47**, 596.

COOKE, L.D., COOKE, T.G., BOOTZ, F. & 4 others (1990). Ploidy as a prognostic indicator in end stage squamous cell carcinoma of the head and neck region treated with cisplatinum. *Br. J. Cancer*, **61**, 759.

COX, D.R. (1972). Regression models and life tables. *J. R. Stat. Soc.*, **34**, 187.

DANES, B.S., BOYLE, P.D., TRAGANOS, F., RINGBORG, U. & MELAMED, M.R. (1987). Evidence of genetic predisposition for some nasopharyngeal cancers by in vitro hyperdiploidy in human dermal fibroblasts. *Cancer Genet. Cytogenet.*, **26**, 261.

ENSLEY, J.F., MACIOROWSKI, Z., HAZZAN, M. & 6 others (1988). Cellular DNA content parameters in untreated and recurrent squamous cell cancers of the head and neck. *Cytometry*, **10**, 334.

FEICHTER, G.E., MAIER, H., ADLER, D. & 4 others (1987). S-phase fractions and DNA ploidy of oropharyngeal squamous epithelium carcinomas compared with histologic grade, stage, response to chemotherapy and survival. *Otolaryngol (Stockh.)*, **104**, 377.

FEINMESSER, R., FREEMAN, J.L. & NOYEK, A. (1990). Flow cytometric anaylsis of DNA content in laryngeal carcinoma. *J. Laryngol. Otol.*, **104**, 485.

FERLITO, A., ANTONUTTO, G. & SILVESTRI, F. (1976). Histological appearances and nuclear DNA content of verrucous squamous cell carcinoma of the larynx. *ORL*, **38**, 65.

FRANZEN, G., OLOFSSON, J., KLINTENBERG, C. & BRUNK, U. (1987*a*). Prognostic value of malignancy grading and DNA measurements in small glottic carcinomas. *ORL*, **49**, 73.

FRANZEN, G., OLOFSSON, J., TYTOR, M., KLINTENBERG, C. & RISBERG, B. (1987*b*). Preoperative irradiation in oral cavity carcinoma. *Acta Oncol.*, **26**, 349.

GOLDSMITH, M.M., CRESSON, D.S., POSTMA, D.S., ASKIN, F.B. & PILLSBURY, H.C. (1986). Significance of ploidy in laryngeal cancer. *Am. J. Surg.*, **152**, 396.

GOLDSMITH, M.M., CRESSON, D.H., ARNOLD, L.A., POSTMA, D.S., ASKIN, F.B. & PILLSBURY, H.C. (1987). DNA flow cytometry as a prognostic indicator in head and neck cancer. *Otolaryngol. Head & Neck Surg.*, **96**, 307.

GRAESSEL-PIETRUSKY, R. & HORNSTEIN, O.P. (1982). Flow cytometric measurement of ploidy and proliferative activity of carcinomas of the oropharyngeal mucosa. *Arch. Dermatol Res.*, **273**, 121.

GUO, Y., DESANTO, L. & OSETINSKY, G.V. (1989). Prognostic implications of nuclear DNA content in head and neck cancer. *Otolaryngol. Head & Neck Surg.*, **100**, 95.

HEDLEY, D.W., FREIDLANDER, M.L., TAYLOR, I.W., RUGG, C.A. & MUSGROVE, E.A. (1983). Method for analysis of cellular DNA content of paraffin-embedded pathological material using flow cytometry. *J. Histochem. Cytochem.*, **31**, 1333.

HEMMER, J. & KREIDLER, J. (1990). Flow cytometric DNA ploidy analysis of squamous cell carcinoma of the oral cavity. *Cancer*, **66**, 317.

HOLM, L. (1982). Cellular DNA amounts of squamous cell carcinomas of the head and neck region in relation to prognosis. *Laryngoscope*, **92**, 1064.

JOHNSON, T.S., WILLIAMSON, K.D., CRAMER, M.M. & PETERS, L.J. (1985). Flow cytometric analysis of head and neck carcinoma DNA index and S-fraction from paraffin embedded sections: comparison with malignancy grading. *Cytometry*, **6**, 461.

KAPLAN, A.S., CALDARELLI, D.D., CHACHO, M.S. & 4 others (1986). Retrospective DNA analysis of head and neck squamous carcinoma. *Arch. Otolaryngol. Head Neck Surg.*, **112**, 1159.

KEARSLEY, H., FURLONG, K.L., COOKE, R.A. & WATERS, M.J. (1990). An immunohistochemical assessment of cellular proliferation markers in head and neck squamous cell cancers. *Br. J. Cancer*, **61**, 821.

KOKAL, W.A., GARDINE, R.L., SHEIBANI, K. & 5 others (1988). Tumour DNA content as a prognostic indicator in squamous cell carcinoma of the head and neck region. *Am. J. Surg.*, **156**, 276.

LAMPE, H.B., FLINT, A., WOLF, G.T. & MCCLATCHEY, K.D. (1987). Flow cytometry: DNA analysis of squamous cell carcinoma of the upper aerodigestive tract. *J. Otolaryngol.*, **16**, 371.

OLINICI, C.D. & CALUSER, I. (1987). DNA content of oral epidermoid carcinomas and of their lymph node metastases. *Morphol. Enbryol.*, **3**, 217.

OLOFSSON, J., FRANZEN, G. & LUNDGREN, J. (1986). Hypertetraploid cells in vocal cord epithelia. *Clin. Otolaryngol.*, **11**, 345.

SICKLE-SANTANELLO, B.J., FARRAR, W.B., DOBSON, J.L., O'TOOLE, R.V. & KEYHANI-ROFAGHA, S. (1986). Flow cytometric analysis of DNA content as prognostic indicator in squamous cell carcinomas of the tongue. *Amer. J. Surg.*, **152**, 393.

STELL, P.M. (1991). Ploidy in head and neck cancer: a review and meta analysis. *Clin. Otol.* (in press).

TYTOR, M., FRANZEN, G. & OLOFSSON, J. (1987). DNA pattern in oral cavity carcinomas in relation to clinical stage and histological grading. *Path. Res. Pract.*, **182**, 202.

TYTOR, M., FRANZEN, G. & OLOFSSON, J. (1989). DNA ploidy in oral cavity carcinomas, with special reference to prognosis. *Head & Neck Surg.*, **11**, 257.

UICC (1987). *TNM Classification of Malignant Tumours*, 4th edition, Geneva.

WILSON, G.D., MCNALLY, N.J., DISCHE, S. & 4 others (1988). Measurement of cell kinetics in human tumours in vivo using bromodeoxyuridine incorporation and flow cytometry. *Br. J. Cancer*, **58**, 423.


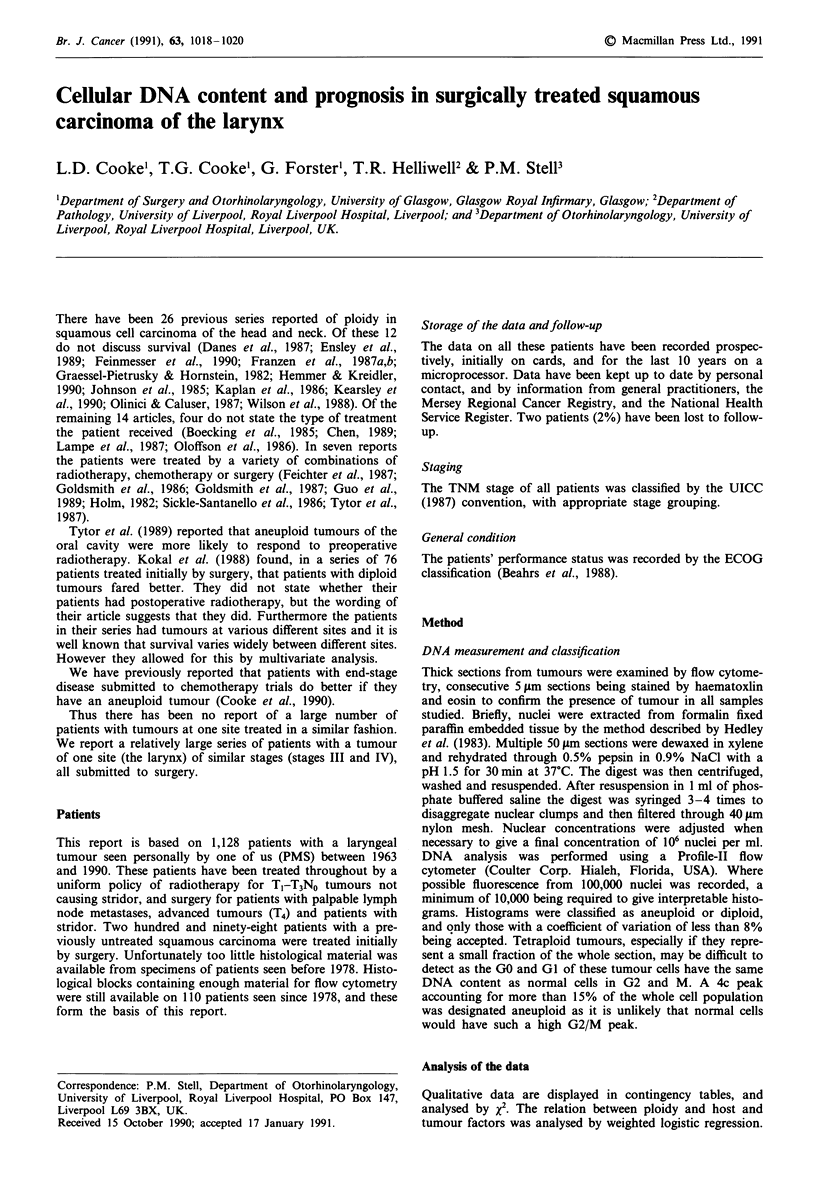

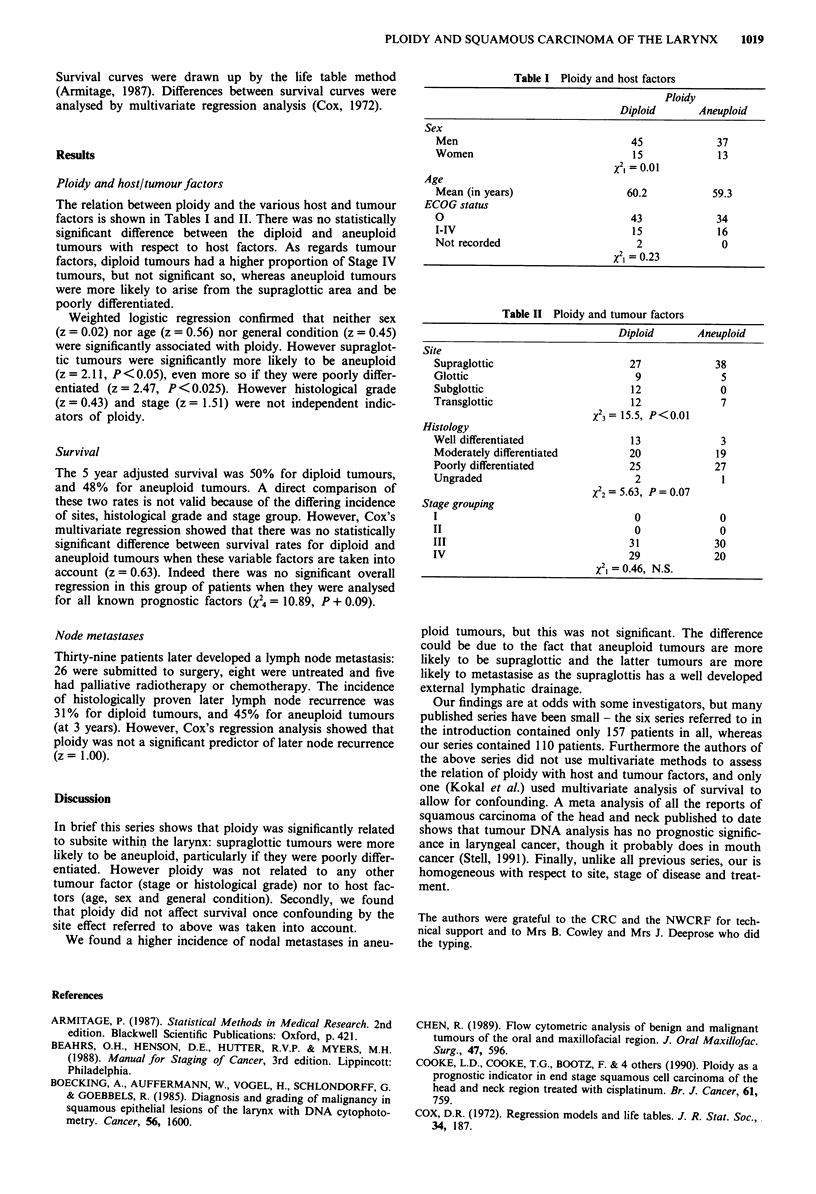

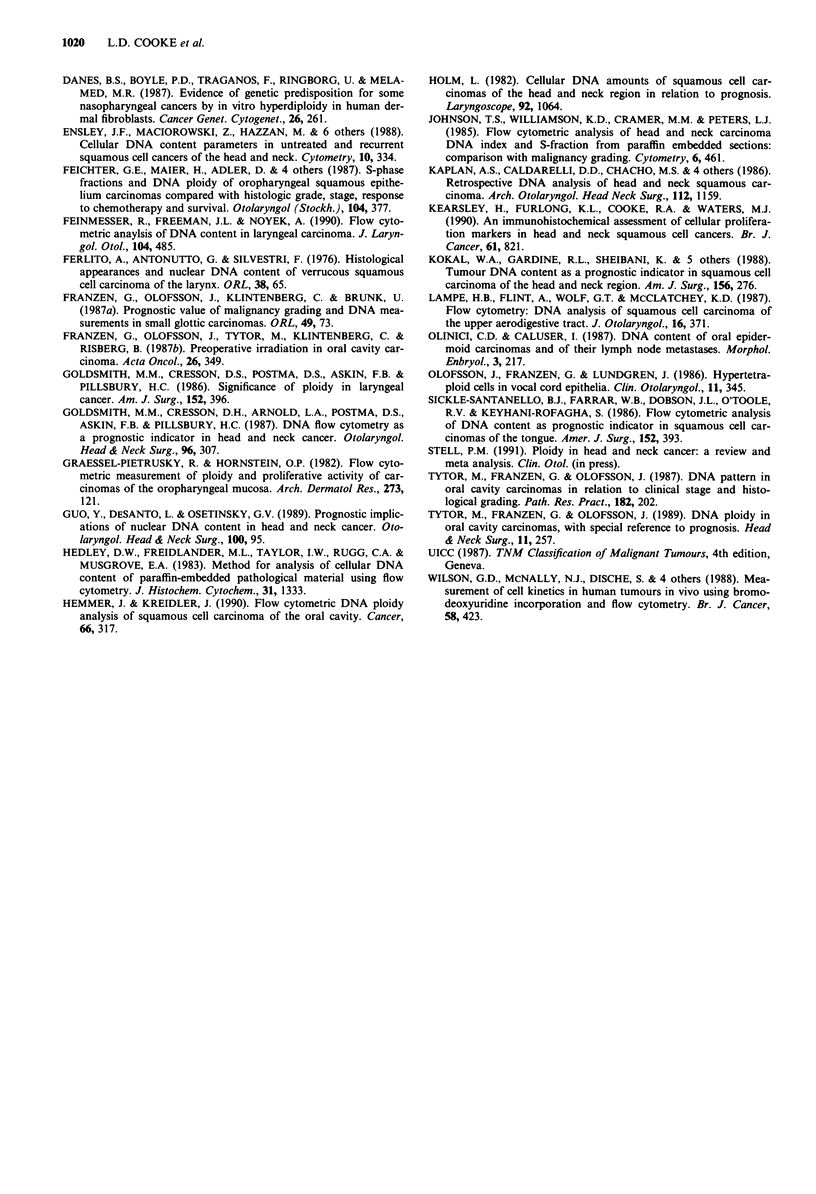


## References

[OCR_00285] Böcking A., Auffermann W., Vogel H., Schlöndorff G., Goebbels R. (1985). Diagnosis and grading of malignancy in squamous epithelial lesions of the larynx with DNA cytophotometry.. Cancer.

[OCR_00291] Chen R. B. (1989). Flow cytometric analysis of benign and malignant tumors of the oral and maxillofacial region.. J Oral Maxillofac Surg.

[OCR_00296] Cooke L. D., Cooke T. G., Bootz F., Forster G., Helliwell T. R., Spiller D., Stell P. M. (1990). Ploidy as a prognostic indicator in end stage squamous cell carcinoma of the head and neck region treated with cisplatinum.. Br J Cancer.

[OCR_00310] Danes B. S., Boyle P. D., Traganos F., Ringborg U., Melamed M. R. (1987). Evidence for genetic predisposition for some nasopharyngeal cancers by in vitro hyperdiploidy in human dermal fibroblasts.. Cancer Genet Cytogenet.

[OCR_00314] Ensley J. F., Maciorowski Z., Hassan M., Pietraszkiewicz H., Heilbrun L., Kish J. A., Tapazoglou E., Jacobs J. R., al-Sarraf M. (1989). Cellular DNA content parameters in untreated and recurrent squamous cell cancers of the head and neck.. Cytometry.

[OCR_00319] Feichter G. E., Maier H., Adler D., Born I. A., Abel U., Haag D., Goerttler K. (1987). S-phase fractions and DNA-ploidy of oropharyngeal squamous epithelium carcinomas compared with histologic grade, stage, response to chemotherapy and survival.. Acta Otolaryngol.

[OCR_00325] Feinmesser R., Freeman J. L., Noyek A. (1990). Flow cytometric analysis of DNA content in laryngeal cancer.. J Laryngol Otol.

[OCR_00330] Ferlito A., Antonutto G., Silvestri F. (1976). Histological appearances and nuclear DNA content of verrucous squamous cell carcinoma of the larynx.. ORL J Otorhinolaryngol Relat Spec.

[OCR_00335] Franzén G., Olofsson J., Klintenberg C., Brunk U. (1987). Prognostic value of malignancy grading and DNA measurements in small glottic carcinomas.. ORL J Otorhinolaryngol Relat Spec.

[OCR_00340] Franzén G., Olofsson J., Tytor M., Klintenberg C., Risberg B. (1987). Preoperative irradiation in oral cavity carcinoma. A study with special reference to DNA pattern, histological response and prognosis.. Acta Oncol.

[OCR_00350] Goldsmith M. M., Cresson D. H., Arnold L. A., Postma D. S., Askin F. B., Pillsbury H. C. (1987). DNA flow cytometry as a prognostic indicator in head and neck cancer.. Otolaryngol Head Neck Surg.

[OCR_00345] Goldsmith M. M., Cresson D. S., Postma D. S., Askin F. B., Pillsbury H. C. (1986). Significance of ploidy in laryngeal cancer.. Am J Surg.

[OCR_00356] Grässel-Pietrusky R., Hornstein O. P. (1982). Flow cytometric measurement of ploidy and proliferative activity of carcinomas of the oropharyngeal mucosa.. Arch Dermatol Res.

[OCR_00362] Guo Y. C., DeSanto L., Osetinsky G. V. (1989). Prognostic implications of nuclear DNA content in head and neck cancer.. Otolaryngol Head Neck Surg.

[OCR_00367] Hedley D. W., Friedlander M. L., Taylor I. W., Rugg C. A., Musgrove E. A. (1983). Method for analysis of cellular DNA content of paraffin-embedded pathological material using flow cytometry.. J Histochem Cytochem.

[OCR_00373] Hemmer J., Kreidler J. (1990). Flow cytometric DNA ploidy analysis of squamous cell carcinoma of the oral cavity. Comparison with clinical staging and histologic grading.. Cancer.

[OCR_00378] Holm L. E. (1982). Cellular DNA amounts of squamous cell carcinomas of the head and neck region in relation to prognosis.. Laryngoscope.

[OCR_00383] Johnson T. S., Williamson K. D., Cramer M. M., Peters L. J. (1985). Flow cytometric analysis of head and neck carcinoma DNA index and S-fraction from paraffin-embedded sections: comparison with malignancy grading.. Cytometry.

[OCR_00389] Kaplan A. S., Caldarelli D. D., Chacho M. S., Bruce D. R., Hutchinson J., Conway S., Coon J. S. (1986). Retrospective DNA analysis of head and neck squamous cell carcinoma.. Arch Otolaryngol Head Neck Surg.

[OCR_00394] Kearsley J. H., Furlong K. L., Cooke R. A., Waters M. J. (1990). An immunohistochemical assessment of cellular proliferation markers in head and neck squamous cell cancers.. Br J Cancer.

[OCR_00400] Kokal W. A., Gardine R. L., Sheibani K., Zak I. W., Beatty J. D., Riihimaki D. U., Wagman L. D., Terz J. J. (1988). Tumor DNA content as a prognostic indicator in squamous cell carcinoma of the head and neck region.. Am J Surg.

[OCR_00405] Lampe H. B., Flint A., Wolf G. T., McClatchey K. D. (1987). Flow cytometry: DNA analysis of squamous cell carcinoma of the upper aerodigestive tract.. J Otolaryngol.

[OCR_00410] Olinici C. D., Căluşer I. (1987). DNA content of oral epidermoid carcinomas and of their lymph node metastases.. Morphol Embryol (Bucur).

[OCR_00415] Olofsson J., Franzén G., Lundgren J. (1986). Hypertetraploid cells in vocal cord epithelia.. Clin Otolaryngol Allied Sci.

[OCR_00419] Sickle-Santanello B. J., Farrar W. B., Dobson J. L., O'Toole R. V., Keyhani-Rofagha S. (1986). Flow cytometric analysis of DNA content as a prognostic indicator in squamous cell carcinoma of the tongue.. Am J Surg.

[OCR_00429] Tytor M., Franzén G., Olofsson J. (1987). DNA pattern in oral cavity carcinomas in relation to clinical stage and histological grading.. Pathol Res Pract.

[OCR_00434] Tytor M., Franzén G., Olofsson J. (1989). DNA ploidy in oral cavity carcinomas, with special reference to prognosis.. Head Neck.

[OCR_00443] Wilson G. D., McNally N. J., Dische S., Saunders M. I., Des Rochers C., Lewis A. A., Bennett M. H. (1988). Measurement of cell kinetics in human tumours in vivo using bromodeoxyuridine incorporation and flow cytometry.. Br J Cancer.

